# ATP Hydrolases Superfamily Protein 1 (ASP1) Maintains Root Stem Cell Niche Identity through Regulating Reactive Oxygen Species Signaling in *Arabidopsis*

**DOI:** 10.3390/plants13111469

**Published:** 2024-05-26

**Authors:** Qianqian Yu, Hongyu Li, Bing Zhang, Yun Song, Yueying Sun, Zhaojun Ding

**Affiliations:** 1 School of Life Sciences, Liaocheng University, Liaocheng 252000, China; lhy20021007@163.com (H.L.); zhangbing5@lcu.edu.cn (B.Z.); songyun@lcu.edu.cn (Y.S.); syysun@aliyun.com (Y.S.); 2 The Key Laboratory of Plant Development and Environmental Adaptation Biology, Ministry of Education, College of Life Sciences, Shandong University, Qingdao 266237, China; dingzhaojun@sdu.edu.cn

**Keywords:** ROS, root stem cell niche, ATP hydrolase, CBSX3

## Abstract

The maintenance of the root stem cell niche identity in *Arabidopsis* relies on the delicate balance of reactive oxygen species (ROS) levels in root tips; however, the intricate molecular mechanisms governing ROS homeostasis within the root stem cell niche remain unclear. In this study, we unveil the role of ATP hydrolase superfamily protein 1 (ASP1) in orchestrating root stem cell niche maintenance through its interaction with the redox regulator cystathionine β-synthase domain-containing protein 3 (CBSX3). ASP1 is exclusively expressed in the quiescent center (QC) cells and governs the integrity of the root stem cell niche. Loss of ASP1 function leads to enhanced QC cell division and distal stem cell differentiation, attributable to reduced ROS levels and diminished expression of *SCARECROW* and *SHORT ROOT* in root tips. Our findings illuminate the pivotal role of ASP1 in regulating ROS signaling to maintain root stem cell niche homeostasis, achieved through direct interaction with CBSX3.

## 1. Introduction

In the whole life of plants, roots play an irreplaceable role that provides a foundation for adapting to various growth environments [1]. The adjustable growth of roots mainly depends on the constantly produced new cells, which are attributed to stem cells dividing around the clock and ultimately differentiating. The root stem cell niche (RSCN) consists of quiescent center (QC) cells and stem cells (SC) around them. QC cells are formed by the lateral division of pituitary cells at the heart-shaped embryo stage. They are totipotent and are the source of stem cells, and cell division is prevented at the G1/S phase, resulting in inactive cell division [2]. Early studies have shown that QC cells can send short-term non-cell-autonomous signals to surrounding stem cells maintaining the undifferentiated state, thereby regulating the maintenance and differentiation of apical stem cells [3,4]. The stem cells around QC can produce diverse tissue cell types through division and differentiation, thereby regulating the growth and development of roots [5]. Current studies suggest that *WOX5* (*WUSCHEL-RELATED HOMEOBOX5*), *SCR* (*SCARECROW*), and *SHR* (*SHORTROOT*) of the GRAS transcription factor family, and the PLETHORA family such as *PLT1* and *PLT2* are key factors that regulate the maintenance of RSCN [6,7,8,9,10,11].

Reactive oxygen species (ROS) are key regulators of plant development [12], containing the root stem cell niche maintenance [13]. To preserve the identification of QC and DSC, the homeostasis of ROS in root tips is crucial. Similar to auxin, either increasing or decreasing ROS levels disturbed the RSCN maintenance, causing an abnormally accelerated QC division and DSC differentiation [14]. Several regulatory factors that are involved in ROS homeostasis to maintain root stem cells have been verified in *Arabidopsis thaliana*. It has been demonstrated that the peptide hormone *RGF1* (Root meristem Growth Factor 1) regulates the new transcription factor *RITF1*, which helps to maintain the root stem cell niche; moreover, the *RGF1* dispersed ROS throughout the root developmental zones, which consequently improved the PLT2 protein’s stability [15]. In addition, Kong reported that PHB3, a prohibitin protein, was involved in determining the identity of the RSCN through affecting the expression of ROS-related genes *NDA* and *NDB* [16]. Additionally, it has been proposed that AtSYP81 (SYNTAXIN OF PLANTS81), a syntaxin/Qa-SNARE protein, plays a role in controlling SCN maintenance via ROS signaling. Furthermore, *PLT1* and *PLT2* were proven to be the downstream factors of AtSYP81 [17]. Phytohormones are essential for regulating cell division and differentiation in SCN. The ABA signaling pathway component *ABO8* (ABA-Overly Sensitive 8) affects the transcription level of *PLT1* and *PLT2* by controlling ROS homeostasis in root tips, creating a connection between ABA, ROS, and PLTs in the maintenance of SCN [18]. The binding of BR to the receptor promotes the increase in intracellular ROS levels, which in turn oxidizes the downstream transcription factor *BZR1* (Brassinazole-Resistant 1) and governs the preservation of the root stem cells [19,20]. Salicylic acid was demonstrated to induce the division of QC cells by increasing levels of reactive oxygen species (ROS) and suppressing the expression of *PLTs* and *WOX5* [21]. All the results emphasized the significance of ROS homeostasis in RSCN maintenance; however, the process by which ROS homeostasis contributes to the identity of root stem cells remains obscure.

ATPases were found to be associated with the homeostasis of ROS in animal cells. HuInd1, a human mitochondrial ATPase, is a crucial factor in the assembly of mitochondrial respiratory chain complex I, which is one of the main sources of mitochondrial ROS production. It was found that huInd1 can bind to the iron–sulfur cluster of mitochondrial complex I (NADH dehydrogenase) in an unstable form through its conserved CXXC binding domain [22]. In addition, a recently discovered obg-like ATPase named *OLA1* acted as an inhibitor of the antioxidant response. The expression of genes encoding antioxidant enzymes did not reveal substantial changes in *OLA1*-knockdown cells [23]. In plants, *APP1*, which belongs to the P-loop NTPase family, was found to cause changes in ROS content in root tips. It was also found that the expression levels of ROS scavenging factors peroxidase genes *PER11* and *PER55* were significantly increased in *app1* mutants. Consistently, the function of mitochondrial complex I was impacted. Nevertheless, the direct regulation mechanism of *APP1* on the above factors is still unclear [14].

In this study, an *asp1* mutant is characterized as showing enhanced QC cell divisions and root DSC differentiation phenotypes, along with a decreased ROS level in the root tip. We reveal that *ASP1* maintains the RSCN identity via regulating ROS homeostasis. *ASP1* is shown to localize within the mitochondria, where it engages in interaction with *CBSX3* (Cystathionine Beta-Synthase X 3), a well-established regulator of reactive oxygen species (ROS) generation in plant mitochondria. Furthermore, we show that *SCR* and *SHR* act downstream of *ASP1*-mediated ROS signaling in the maintenance of RSCN identity.

## 2. Results

### 2.1. ASP1 Plays a Role in ROS Maintenance to Control RSCN Identity

**Figure 1:**
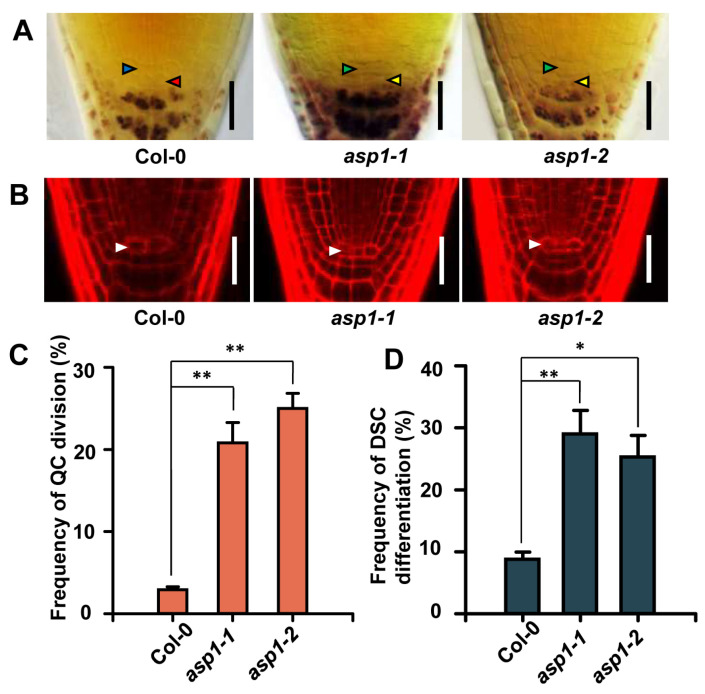
The *asp1* mutants show defects in root SCN maintenance. (**A**) Staining with Lugol’s solution was performed on the roots of 5-day-old Col-0 and *asp1* mutants. The enhanced rate of divided QC cells and differentiated distal stem cells was revealed by Lugol staining in *asp1* mutants. Regular QC cells are represented by blue arrows, standard distal stem cells are represented by red arrows, divided QC cells are represented by green arrows, and abnormal distal stem cells are represented by yellow arrows. The scale bars are 50 μm in length. (**B**) Mutation of *ASP1* resulted in QC cell division indicated by confocal microscope scanning images of PI staining. QC is marked with white arrows. The scale bars are 50 μm in length. (**C**,**D**) Measuring the number and analyzing the frequency of divided QC cells and differentiated distal stem cells in WT and *asp1*. The data presented show the average values with standard error (*n* = 100), with significance levels indicated as * for *p* < 0.05 and ** for *p* < 0.01. The statistical analysis used was Student’s *t*-test.

**Figure 2:**
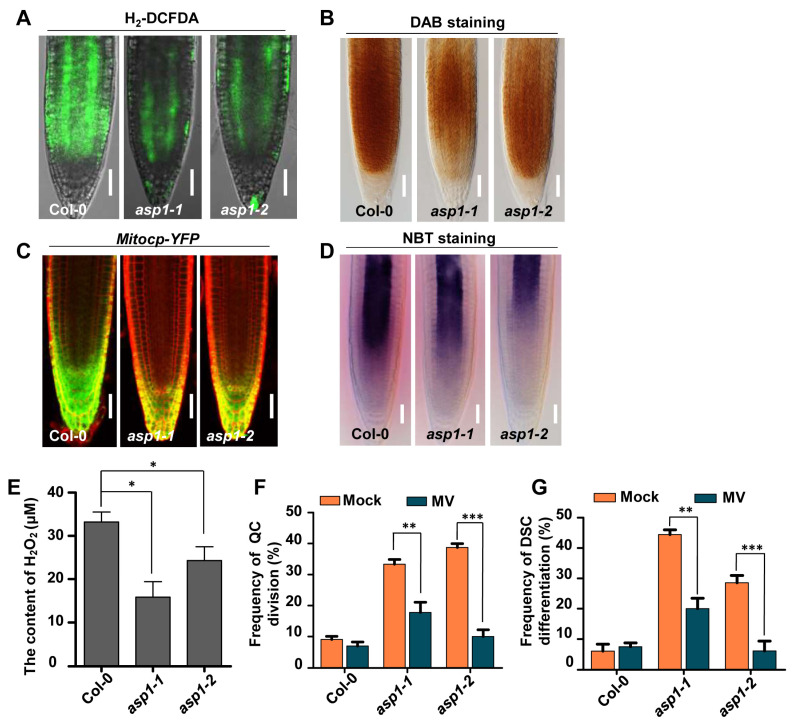
The defects of SCN maintenance in *asp1* mutants were attributed to the lowered ROS level. (**A**) Fluorescent examination of H_2_-DCFDA in the roots of Col-0 and *asp1* was conducted. The scale bars represent 50 μm. (**B**) DAB staining of 5-day-old seedlings. The scale bars represent 50 μm. (**C**) Detection of the fluorescence signal from *Mitocp-YFP* in Col-0 and *asp1* mutants. The scale bars represent 50 μm. (**D**) NBT staining of 5-day-old seedlings. The scale bars represent 50 μm. (**E**) Measurement of endogenous levels of H_2_O_2_ in Col-0 and *asp1* roots. (**F**, **G**) Quantification of divided QC cells and differentiated distal stem cells in Col-0 and *asp1* roots treated with or without 0.1 µM MV. Results are presented as averages with standard error (*n* = 100), with significance levels denoted as * for *p* < 0.05, ** for *p* < 0.01, *** for *p* < 0.001. Statistical analysis was conducted using Student’s *t*-test.

A genetic screening was carried out on *Arabidopsis* mutants to discover new elements associated with the maintenance of root stem cells regulated by ROS. This screening aimed to identify mutants exhibiting impairments in root distal stem cell maintenance and alterations in ROS levels within a mutant pool generated through the insertion of T-DNA [24]. From Lugol’s staining and H_2_-DCFDA staining, the *asp1-1* mutant (Salk_036792) was identified, which displayed increased QC cell divisions and distal stem cell differentiation along with a decreased ROS level in the root tip (Figure 1A and Figure 2A).

Using PI staining (for red fluorescence), the dividing QC cells were observed in the *asp1-1* mutant other than the WT (wild type) under a confocal microscope (Figure 1B). In the wild type, less than 5% of QC cells were divided (Figure 1C); however, the frequency of divided QC cells in the *asp1-1* mutant was more than 20% (Figure 1C). Furthermore, the *asp1-1* mutant showed a higher proportion of DSC differentiation (around 30%), contrary to the low rate in the WT (around 5%) (Figure 1D). Consequently, these results demonstrate that the loss of function of *ASP1* results in developmental defects in RSCN.

Tail-PCR analysis showed that the last exon in the *asp1-1* (*At4g27680*) allele is inserted with a T-DNA fragment (Figure S1A). Another mutant *asp1* allele (*asp1-2*, *Sail_547_g04*) was analyzed to prove this mutation, as the *asp1-2* mutant showed the same phenotype as *asp1-1* (Figure 1 and Figure S1B). Moreover, the expression of *ASP1* was significantly reduced in both mutants (Figure S1B). The defects of root SCN maintenance in the *asp1-1* mutant were complemented through crossing with the *ASP1p::GFP-ASP1* transgenetic line (Figure S1C). Taken together, these findings provide evidence to show that the observed phenotype in *asp1* mutants can be attributed to the mutation in *ASP1*.

To further confirm the decreased ROS contents in *asp1* mutants as indicated by H_2_-DCFDA, we examined the extent of hydrogen peroxide (H_2_O_2_) and superoxide anion (O^2−^), the two primary kinds of ROS in plants (Figure 2). Compared to WT, the level of H_2_O_2_ (indicated by DAB staining) and O^2−^ (indicated by NBT staining) in the *asp1* mutants was reduced (Figure 2B,D). When crossed with *Mitocp-YFP*, a mitochondrial O^2−^ indicator, a weaker signal was observed in the *asp1* mutant roots compared to controls (Figure 2C). In addition, the contents of H_2_O_2_ were greatly declined in *asp1* mutants (Figure 2E). Meanwhile, *APX2*, *APX3*, *APX4*, and *APX6*—which encode ROS-scavenging enzymes—were up-regulated in the *asp1-1* mutant (Figure S2). These findings indicate *ASP1* plays a role in regulating the maintenance of ROS levels.

To test the impact of altered ROS levels in *asp1* mutants on the defects in root SCN maintenance, Col-0 and *asp1* mutants were treated with methyl viologen (MV), a compound known to induce an excess production of superoxide. Different from the WT, the elevated occurrence of division in QC and differentiation in DSC were significantly decreased after treatment with MV in *asp1* mutants (Figure 2F,G). These recovered SCN-defective phenotypes in *asp1* mutants might be associated with the elevated ROS contents induced by MV. Moreover, the enhanced frequency of abnormal QC and root DSC in ASP1 over-expression lines was attributed to the elevated ROS level (Figure S3). Taken together, we conclude that ASP1 controls RSCN identity through regulating ROS homeostasis.

### 2.2. ASP1 Is Exclusively Expressed in QC Cells and Localized in Mitochondria

**Figure 3:**
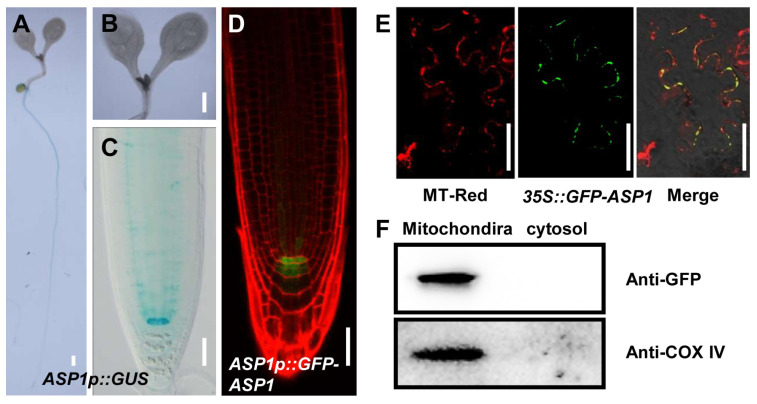
Patterns of expression and cellular localization of *ASP1*. (**A**–**C**) Histochemical staining analysis of *ASP1p::GUS* transgenic lines. (**A**) GUS staining of whole seedlings of 5-day-old *ASP1p::GUS* transgenic line. Scale bar: 10 mm. (**B**) Magnification of the leaf of *ASP1p::GUS* transgenic line in (**A**). Scale bar: 10 mm. (**C**) Magnification of the leaf and root tips of *ASP1p::GUS* transgenic line in (**A**). Scale bar: 50 μm. (**D**) Image captured using confocal microscopy showing *ASP1p::GFP-ASP1* in root tips, with GFP fluorescence in green and PI fluorescence in red. The scale bar represents 50 μm. (**E**) The signal of *GFP-ASP1* is found in the same location as the Mito Tracker Red in tobacco. The scale bar represents 50 μm. (**F**) Western blotting analysis of mitochondria and cytosol proteins isolated from *35S::GFP-ASP1* seedlings. COX IV was utilized as a reference standard in this examination.

To clarify the probable effect of ASP1 in ROS-modulated root SCN maintenance, we constructed *ASP1p::GUS* and *ASP1p::GFP-ASP1* transgenic lines for the purpose of examining the expression profile of ASP1. GUS staining analysis with *ASP1p::GUS* lines indicates that ASP1 is specifically expressed in QC cells (Figure 3A–C). Consistently, strong GFP signals were detected in the root QC cells through analysis of *ASP1p::GFP-ASP1* transgenic lines (Figure 3D).

In addition, we further generated a *35S::GFP-ASP1* construct to study the subcellular localization of ASP1. The results observed under a confocal microscope showed that the green fluorescence signal of ASP1 and the red fluorescence signal of Mito Tracker Red (mitochondrial probe) were largely merged (Figure 3E), indicating ASP1 is localized in mitochondria. To further confirm the mitochondrial localization of ASP1, we isolated mitochondria and cytosol components (without mitochondria) from the *35S::GFP-ASP1* transgenic line. Western blotting analysis detected the ASP1-GFP protein and COX IV; (mitochondria marker protein) from mitochondria proteins other than cytosol components (without mitochondria) (Figure 3F). To explore whether ASP1 is located in other endomembrane systems, a specific antibody marker for endoplasmic reticulum (ER)-associated BIP2 was imported. Immunologic analysis showed minimal co-localization between the signals of GFP-ASP1 and BIP2 (Figure S4).

### 2.3. SCR and SHR Are Implicated in ASP1-Mediated ROS Homeostasis Regulating the SCN Maintenance

**Figure 4:**
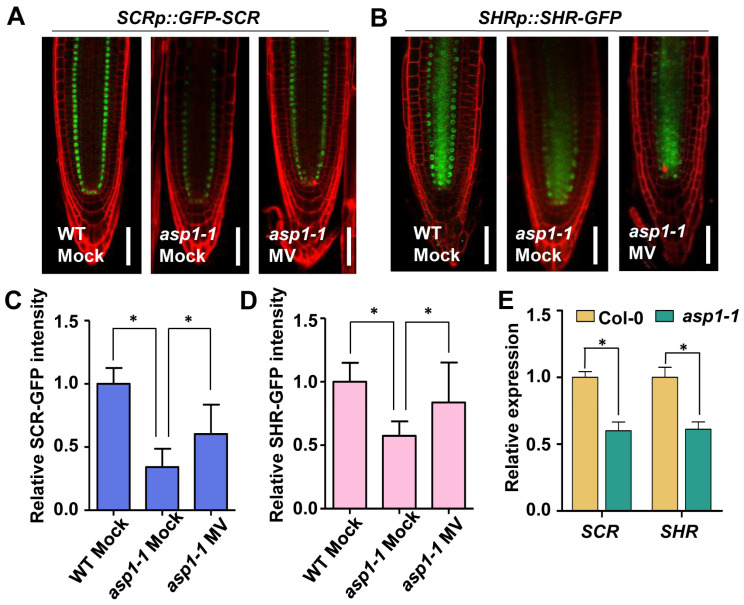
SCR and SHR act downstream of *ASP1* and respond to ROS homeostasis to regulate RSCN maintenance. (**A**) Fluorescence analysis of *SCRp::GFP-SCR* in Col-0 and *asp1* roots treated with or without MV treatment. The scale bar represents 50 μm. (**B**) The fluorescence signal detection of *SHRp::SHR-GFP* in Col-0 and *asp1* mutants roots with or without MV treatment. The scale bar represents 50 μm. (**C**,**D**) Measurement of the intensity of GFP in *SCRp::GFP-SCR* and *SHRp::GFP-SHR* seedlings in (**A**,**B**) with or without 0.1 µM MV. Results are presented as averages with standard error (*n* = 30), with statistical significance denoted by * *p* < 0.05 using Student’s *t*-test. (**E**) qRT-PCR results show the relative transcript levels of SCR and SHR in Col-0 and *asp1-1* mutant. Results are presented as averages with standard error (*n* = 3), with statistical significance denoted by * *p* < 0.05 using Student’s *t*-test.

To investigate whether the transcription factors important for root SCN maintenance are involved in the ASP1 regulatory pathway, we detected the expression levels of WOX5, PLT1, PLT2, as well as SCR and SHR in the WT and *asp1* mutants. The real-time PCR examination indicated that there were no significant differences in the expression levels of WOX5, PLT1, and PLT2 between the *asp1* mutants and the wild type (Figure S5A). Similarly, there was no difference in the fluorescence signals of *WOX5p::GFP*, *PLT1p::PLT1-YFP,* and *PLT2p::PLT2-YFP* between Col-0 and *asp1* mutants (Figure S5B). However, the levels of expression for SHR and SCR were notably decreased in *asp1* mutants, as evidenced by fluorescence examinations using *SCRp::GFP-SCR* and *SHRp::SHR-GFP* (Figure 4A–D). The analysis of qRT-PCR revealed a consistent decrease in the expression levels of SHR and SCR in *asp1* mutants. This suggests that the down-regulation of SCR and SHR may be involved in the ASP1-mediated regulation of RSCN maintenance (Figure 4E).

Additionally, we explored whether the reduced SCR and SHR expression in *asp1* was related to the decreased ROS level. We tested the effect of methyl viologen on SCR and SHR in the *SCRp::GFP-SCR* and *SHRp::SHR-GFP* transgenetic lines in the *asp1* mutant background. The findings indicated a significant elevation in GFP signals within root tips after methyl viologen treatment (Figure 4A–D), suggesting that ASP1 plays a role in regulating ROS levels to preserve the root stem cell niche via the SCR and SHR pathways.

### 2.4. ASP1 Interacts Directly with CBSX3 to Maintain Root SCN Stability via Manipulating ROS Generation

**Figure 5:**
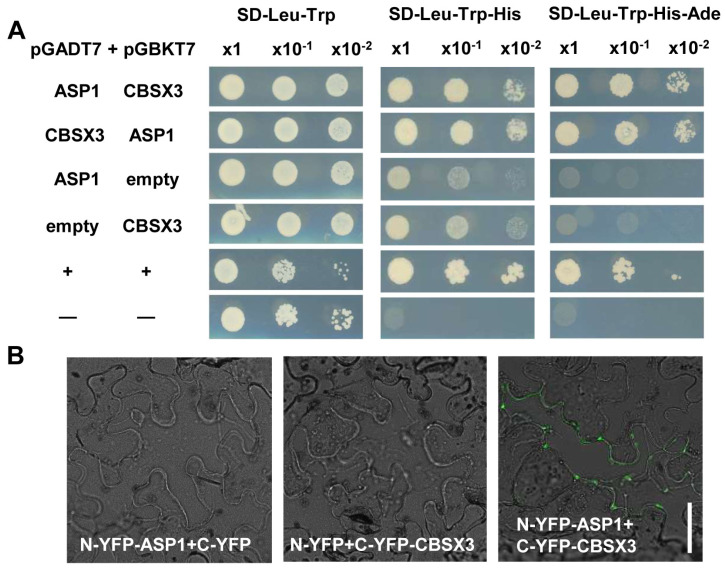
ASP1 interacts with CBSX3. (**A**) The identification of the interaction between ASP1 and CBSX3 by using yeast. Co-transformed cells were plated on SD–Leu–Trp agar to assess their survival and on SD–Leu–Trp–His and SD–Leu–Trp–His–Ade agars to evaluate their interaction. (**B**) BiFC analysis was conducted to study the interaction between ASP1 and CBSX3. Different combinations of constructs and empty vectors were introduced into tobacco, and then observe the fluorescence signal via confocal microscope. The scale bar on the images was set at 50 μm.

To uncover how ASP1 regulates ROS homeostasis, a yeast two-hybrid assay was conducted to elucidate the protein interactions of ASP1, leading to the identification of a CBS domain-containing protein, CBSX3 (Figure 5A). The association of ASP1 and CBSX3 was additionally validated through a bimolecular fluorescence complementation (BiFC) assay. In tobacco epidermal cells, a pronounced fluorescence signal was detected when ASP1 was coexpressed with the N-terminal half of YFP (NYFP), and CBSX3 was co-expressed with the C-terminal half of YFP (CYFP). Conversely, no fluorescence signal was detected in the control group where the empty vector was utilized (Figure 5B). All these results indicate that there is an interaction between ASP1 and CBSX3.

**Figure 6:**
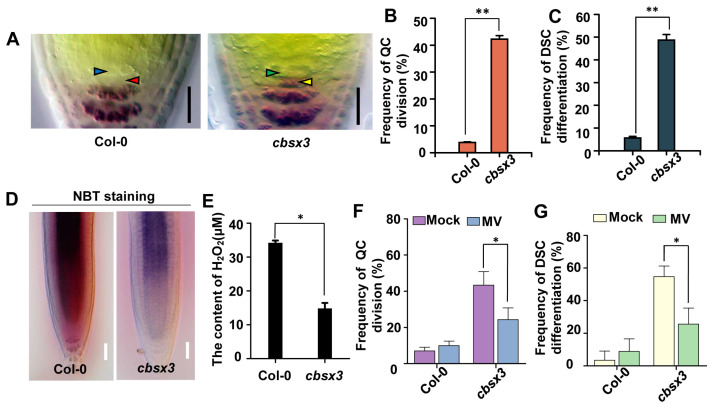
Mutation of *CBSX3* affects the root stem cell niche identity via regulating ROS homeostasis. (**A**) Lugol-staining roots of Col-0 and *cbsx3* mutants. Blue arrow indicates regular QC, red arrow indicates the undifferentiated DSC layers, green arrow indicates dividing QC, and yellow arrow indicates the differentiated distal stem cells. Scale bars are 50 μm in length. (**B**,**C**) Quantification of divided QC cells and differentiated distal stem cells in Col-0 and *cbsx3*. (**D**) Staining with NBT was performed on seedlings of Col-0 and *cbsx3* that were 5 days old. Scale bars represent 50 μm. (**E**) Measurement of H_2_O_2_ content in roots of Col-0 and *cbsx3*. (**F**,**G**) The frequency analysis of divided QC cells and differentiated distal stem cells in Col-0 and *cbsx3* seedlings with or without MV treatment. Results are presented as averages with standard error (*n* = 100), with significance levels denoted as * for *p* < 0.05, ** for *p* < 0.01. Statistical analysis was conducted using Student’s *t*-test.

CBSX3 has been reported to regulate ROS generation and CBSX3-Ox plants display primary root shortening. Nevertheless, the impact of CBSX3 on the RSCN remains unexplored. Consequently, we investigated the characteristics of the root quiescent center cells and distal stem cells in *cbsx3* (Figure 6A). In the *cbsx3* mutant, a significant increase in QC cell division rate was observed, exceeding 40% in contrast to the wild-type frequency of 5%. Moreover, approximately half of the mutant roots exhibited a lack of distal stem cells, indicating the potential involvement of CBSX3 in the maintenance of the RSCN (Figure 6B,C). To investigate the role of CBSX3 in maintaining root RSCN integrity through the regulation of ROS homeostasis, we conducted an analysis of ROS levels in the roots of *cbsx3*. In comparison to the wild type, reduced NBT staining intensity was noted in the *cbsx3* root tips (Figure 6D). Furthermore, the concentration of hydrogen peroxide was found to be reduced in *cbsx3* in comparison to the wild type (Figure 6E). Therefore, impairment of CBSX3 results in a decrease in ROS generation in the root tips.

To further explore the potential relationship between the increased percentage of divided QC cells, differentiated distal stem cells in *cbsx3* mutants, and the reduced levels of ROS, the root stem cell niche was examined after being supplied with methyl viologen. Strikingly, the elevated frequency was strongly decreased after 0.1 μM MV treatments (Figure 6F,G). These results indicate that CBSX3 acts as a controller of reactive oxygen species generation, thereby influencing the root stem cell niche identity.

## 3. Discussion

**Figure 7:**
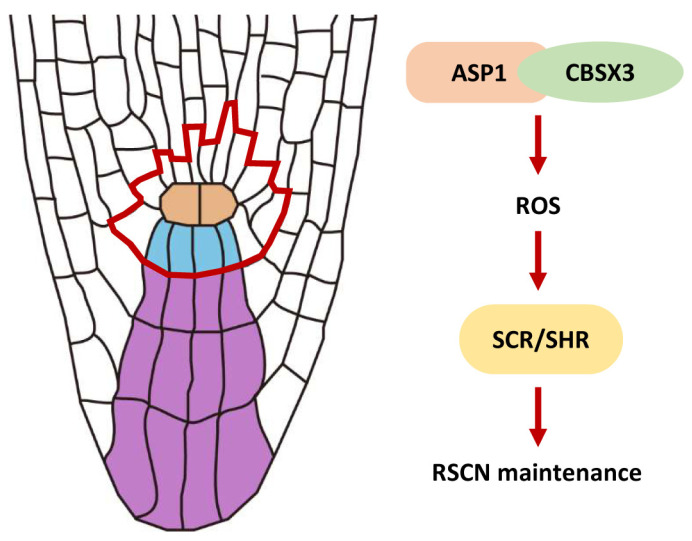
*ASP1*-mediated ROS homeostasis to regulate RSCN maintenance. *ASP1* regulates ROS signaling through interacting with the redox regulator *CBSX3*. Furthermore, *SCR* and *SHR* act downstream of *ASP1*-mediated ROS signaling in the maintenance of RSCN identity.

The AAA ATPase family is a large and functionally diverse group of enzymes that are involved in numerous cellular processes [25]. All ATPases in this family have a domain of 200–250 amino acids that is responsible for binding ATP [26]. AAA ATPases can be found in all eukaryotic organisms, with the highest diversity in *Arabidopsis*, with about 140 AAA^+^ proteins [27]. AAA ATPases play a significant role in controlling various developmental processes in plants, including but not limited to embryonic development, pollen formation, seed development, and the regulation of flowering time [28,29,30,31]; moreover, AAA ATPases play a role in the control of the root meristem. As an illustration, RPT2 maintains the root meristem integrity via proteasome-dependent programmed proteolysis [32]; the function of BRM is to preserve the RSCN by regulating the expression of PIN proteins within the PLT signaling [33]; and MDN1 is essential for the auxin maxima pattern in the embryo and functions in root meristem maintenance [34]. However, the involvement of AAA ATPases in the control of ROS homeostasis for the maintenance of RSCN has not been described. In this study, we provide evidence to show that ASP1, a novel AAA ATPase, maintains the RSCN by manipulating ROS generation in the root tip (Figure 1 and Figure 2). Therefore, our findings broadened our understanding of AAA ATPase function and ROS homeostasis regulation (Figure 7).

Research has demonstrated the significant involvement of ROS homeostasis in the maintenance of the RSCN [13]. The altered ROS levels lead to defects in root SCN maintenance in both *asp1* and *cbsx3* mutants (Figure 2, Figure 6, and Figure S3). Our findings, together with earlier studies, indicate that maintaining a suitable level of ROS is crucial for preserving the RSCN [14]; however, the molecular mechanism of ROS homeostasis is finely regulated to preserve the identity of RSCN remains unknown. In this research, we present findings that ASP1 interacts with a ROS generation regulator CBSX3 to regulate the homeostasis of ROS (Figure 5 and Figure 6). The CBS (cystathionine β-synthase) domain-containing proteins are a large superfamily of proteins containing a conserved CBS domain [35]. CBSX3 was reported to localize to the mitochondria, and has the same subcellular localization as ASP1 (Figure 3). A previous study showed that CBSX3 regulates ROS generation by interacting with Trxs-o2 in an ADP/ATP ratio-dependent manner [36]. Further studies are needed to explore how ASP1 regulates ROS homeostasis through interacting with CBSX3; therefore, it will also be interesting to study whether ASP1 could affect the interaction between CBSX3 and Trxs-o2 by altering the ADP/ATP ratio.

SCR and SHR, members of GRAS transcription factors, play a crucial role in RSCN maintenance [37]. Both *scr* and *shr* mutants display defective QC and DSC maintenance and lead to short-root phenotypes [38,39]. After the synthesis of SHR in the vascular cylinder, it is translocated to the neighboring cell layer to activate SCR [40]. In addition, SCR interacts with PLT and TCP, which is short for teosinte-branched cycloidea PCNA, to determine the specific location of stem cells. These complexes have the ability to stimulate the activation of WOX5 expression, a gene that is uniquely expressed in QC and plays a crucial role in maintaining RSCN integrity, by binding to the WOX5 promoter [9]. A previous study demonstrated that SCR and SHR respond to ROS homeostasis to regulate root DSC maintenance [14]. This report is consistent with our finding that the expression of SCR and SHR was suppressed in *asp1* mutants and could be increased by MV (Figure 4 and Figure S5). Based on these observations, we propose that SCR and SHR are involved in ASP1-mediated ROS homeostasis regulating the maintenance of SCN. Further experiments are required to establish the specific correlation between ASP1 and the SCR/SHR pathway.

## 4. Materials and Methods

### 4.1. Plant Materials and Growth Conditions

The Arabidopsis mutants and transgenic lines employed in this investigation are derived from the ecotype Columbia (Col-0). The *asp1-1* (SALK_036792) and *asp1-2* (SAIL_547_G04) were T-DNA insertion mutants acquired from the Arabidopsis Biological Resource Center. The marker lines were previously discussed in other sources: *Mito-cpYFP* [41], *SCRp::GFP-SCR* [38], *SHRp::SHR-GFP* [42], *WOX5p::GFP* [5], *PLT1p::PLT1-YFP* [43], *PLT2p::PLT2-YFP* [43]. The *ASP1pro::GUS* fusion was generated through the insertion of a 1300-base-pair sequence of the ASP1 promoter region into pKGWFS7.1. The sequences of the ASP1 (AT4G27680) promoter and coding region were integrated into pB7m34GW to acquire the *ASP1pro::GFP-ASP1* fusion. The CDS of ASP1 was integrated into pK7WGF2.0 to obtain the *35S::GFP-ASP1* fusion. For MV treatment, 3-day-old seedlings were incubated with 0.1 μM MV in a 1/2 liquid MS medium for 48 h. Seeds were subjected to surface sterilization using chlorine gas and subsequently stored at 4 °C for a period of two days. Following this, the seeds were placed onto solid medium containing half-strength Murashige and Skoog (MS) nutrients and subsequently transferred to a plant growth chamber set at a constant temperature of 20 °C under a 16-h photoperiod.

### 4.2. Histochemical Assays and Microscopy

To perform Lugol’s staining, the seedlings were steeped in Lugol’s solution for 2 min, then placed in chloral hydrate solution and imaged by a phase-contrast microscope. The H_2_-DCFDA (Invitrogen, Carlsbad, CA, USA) staining assay was carried out following the previously outlined procedure [44], and then photographed by a laser-scanning confocal microscope (excitation: 488 nm, emission: 525 nm). Five-day-old seedlings were placed in a solution of DAB (Sigma, St. Louis, CA, USA) containing 0.1 mg/mL DAB in a solution of 50 mM Tris-HCl and NBT (Sigma) containing 2 mM NBT in a solution of 0.1 M NaCl and 20 mM potassium phosphate, with a pH of 6.1, for 15 min. Subsequently, they were observed using a light microscope. For measurement of H_2_O_2_ content, roots isolated from five-day-old WT and *asp1* seedlings were ground with extraction buffer (Beyotime Biotechnology, Shanghai, China) on ice, and measured at 560 nm by a spectrophotometer.

### 4.3. Western Blotting

The proteins from the mitochondria and cytosol of *35S::GFP-ASP1* roots were separated using the mitochondrial protein extraction kit from Solarbio (Beijing, China). The proteins were separated using SDS-PAGE (Bio-Rad, Hercules, CA, USA) and subsequently moved onto PVDF membranes (Millipore, St. Louis, CA, USA). The membranes were first treated with 5% milk for 2 h, then exposed to GFP and COX IV antibodies overnight at 4 °C, and finally incubated with secondary antibodies conjugated with horseradish peroxidase for 2 h. The SuperSignal West Pico Luminol Solution (ThermoFisher Scientific, Carlsbad, CA, USA) detected the signal.

### 4.4. Quantitative RT-PCR

The RNA template required was obtained by isolating it using an RNAsimple Total RNA Kit from Tiangen. Next, an initial cDNA strand was created from 2 μg of RNA by utilizing a HiScript III RT SuperMix for qPCR from Vazyme (Nanjing, China), then, DNase I was used to eliminate genomic DNA. QRT-PCRs were conducted on the MyiQTM Real-time PCR Detection System from Bio-Rad, utilizing the AceQ Universal SYBR qPCR Master Mix from Vazyme. Each sample consisted of three biological replicates, with each biological replicate being represented by three technical replicates. The reference gene used was AtACTIN2. The quantitative RT-PCR primers are detailed in the Supplementary Table S1.

### 4.5. Yeast Two-Hybrid Assay

This assay was conducted following the instructions provided in the manual from Clontech for the Matchmaker GAL4 Two-Hybrid System 3. The coding sequences of ASP1 and CBSX3 were placed into the pGADT7 vector from Takara and separately cloned into the pGBKT7 vector. The modified yeasts were grown on SD–Trp–Leu medium for 2 days before being moved to SD–Trp–Leu–His medium.

### 4.6. BiFC Assay

The fragment of ASP1 was amplified and inverted into the pSAT6-nYFP, and the CDS of CBSX3 was inserted into the pSAT6-cYFP. The fusions were injected into *Nicotiana benthamiana* leaves. Signals were observed after culturing at 25 °C for 3 days by using a Zeiss LSM 700 confocal microscope. The blank vector was utilized as a negative control.

## Data Availability

All data are available in the main text or the Supplementary Materials.

## Supplementary Materials

The following supporting information can be downloaded at: https://www.mdpi.com/article/10.3390/plants13111469/s1, Figure S1: the validation of *asp1* mutants; Figure S2: quantitative real-time PCR was utilized to assess the comparative transcript levels of ROS generation and scavenging genes; Figure S3: the increased ROS concentration observed in ASP1-OE line stimulates the division of QC and differentiation of DSC; Figure S4: the subcellular distribution of ASP1 was examined, revealing minimal overlap between the signal of GFP-ASP1 and the endoplasmic reticulum (ER) marker BIP2; Figure S5: expression of WOX5, PLT1, and PLT2 in *asp1* mutants did not show a statistically significant variance compared to those observed in the Col. Table S1: List of primers used in the study.
