# Supplementary material for: ATP Hydrolases Superfamily Protein 1 (ASP1) Maintains Root Stem Cell Niche Identity through Regulating Reactive Oxygen Species Signaling in Arabidopsis
Source: Plants (Basel). 2024 May 26;13(11):1469. doi: 10.3390/plants13111469 (PMC11174532; doi:10.3390/plants13111469)
Supplement: Supplementary file 1 [file plants-13-01469-s001.zip › Supplemental Figures.pdf]

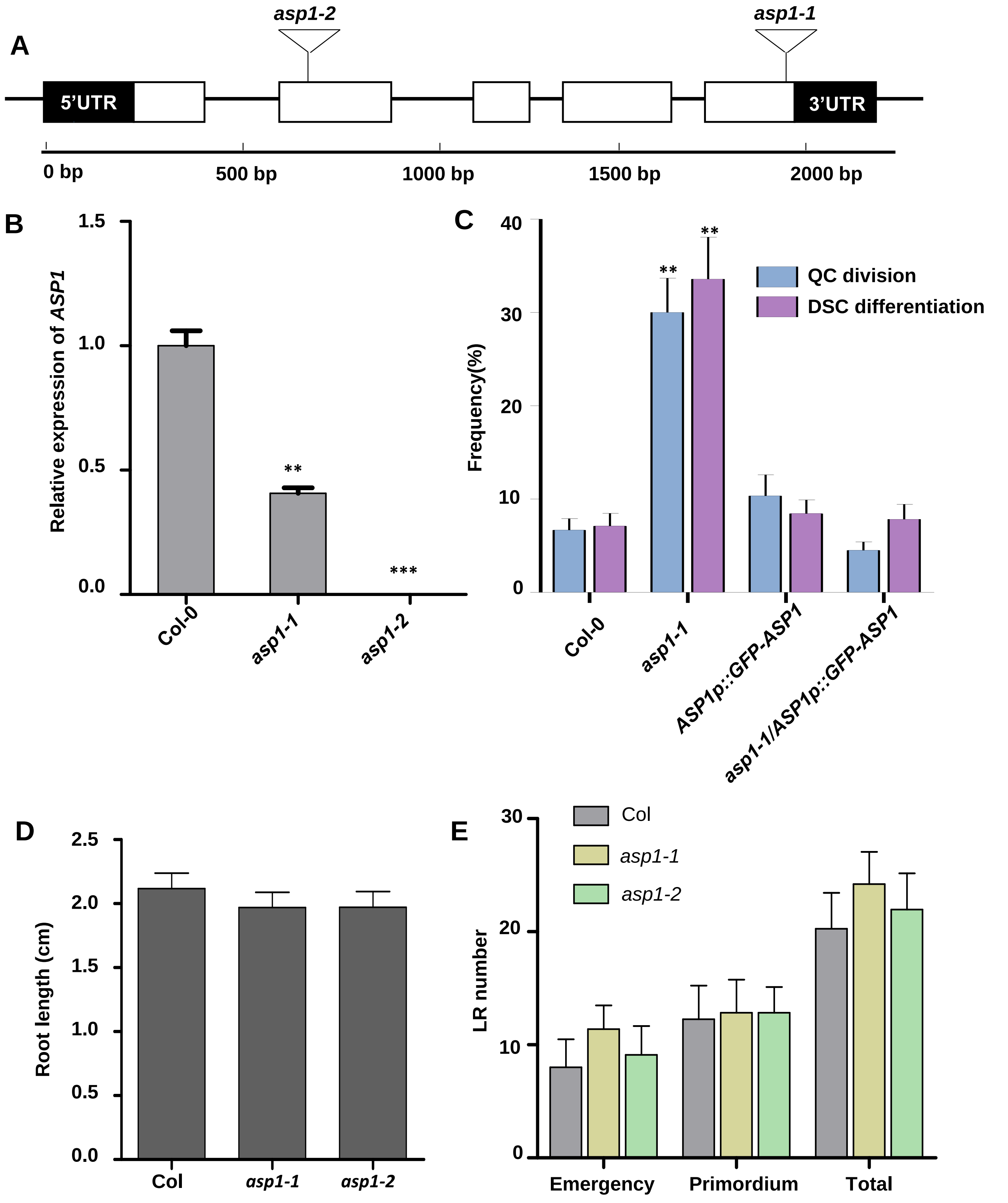
A
asp1-2
asp1-1
5'UTR
3'UTR
0 bp
500 bp
1000 bp
1500 bp
2000 bp
B
Relative expression of ASP1
1.5
1.0
0.5
0.0
**
***
Col-0
asp1-1
asp1-2
C
Frequency(%)
40
30
20
10
0
**
**
QC division
DSC differentiation
Col-0
asp1-1
ASP1p::GFP-ASP1
asp1-1/ASP1p::GFP-ASP1
D
Root length (cm)
2.5
2.0
1.5
1.0
0.5
0.0
Col
asp1-1
asp1-2
E
LR number
30
20
10
0
Col
asp1-1
asp1-2
Emergency
Primordium
Total

Figure S1. The validation of *asp1* mutants. (A) The locations of T-DNA insertions in the mutants *asp1-1* and *asp1-2* are depicted. Exons are denoted by boxes, while introns are represented by lines. (B) Quantitative real-time PCR was utilized to assess the comparative transcript levels of *ASP1* in both wild-type and *asp1*. The results are presented as mean values with standard error (n = 3), where statistical significance is denoted as * for $p < 0.05$, ** for $p < 0.01$, and *** for $p < 0.001$. (C) Quantification of divided QC cells and differentiated distal stem cells in Col, *asp1* mutant, *ASP1p::GFP-ASP1* and *asp1-1/ASP1p::GFP-ASP1*. The results are presented as mean values with standard error (n = 100), and statistical significance was determined at a level of $p < 0.01$. (D) The length of primary root of five-day-old Col and *asp1* mutants. The results are presented as mean values with standard error (n = 30). (E) The number of lateral root of 10-day-old Col and *asp1* mutants. The results are presented as mean values with standard error (n = 30). The statistical analysis used was a Student's *t*-test.

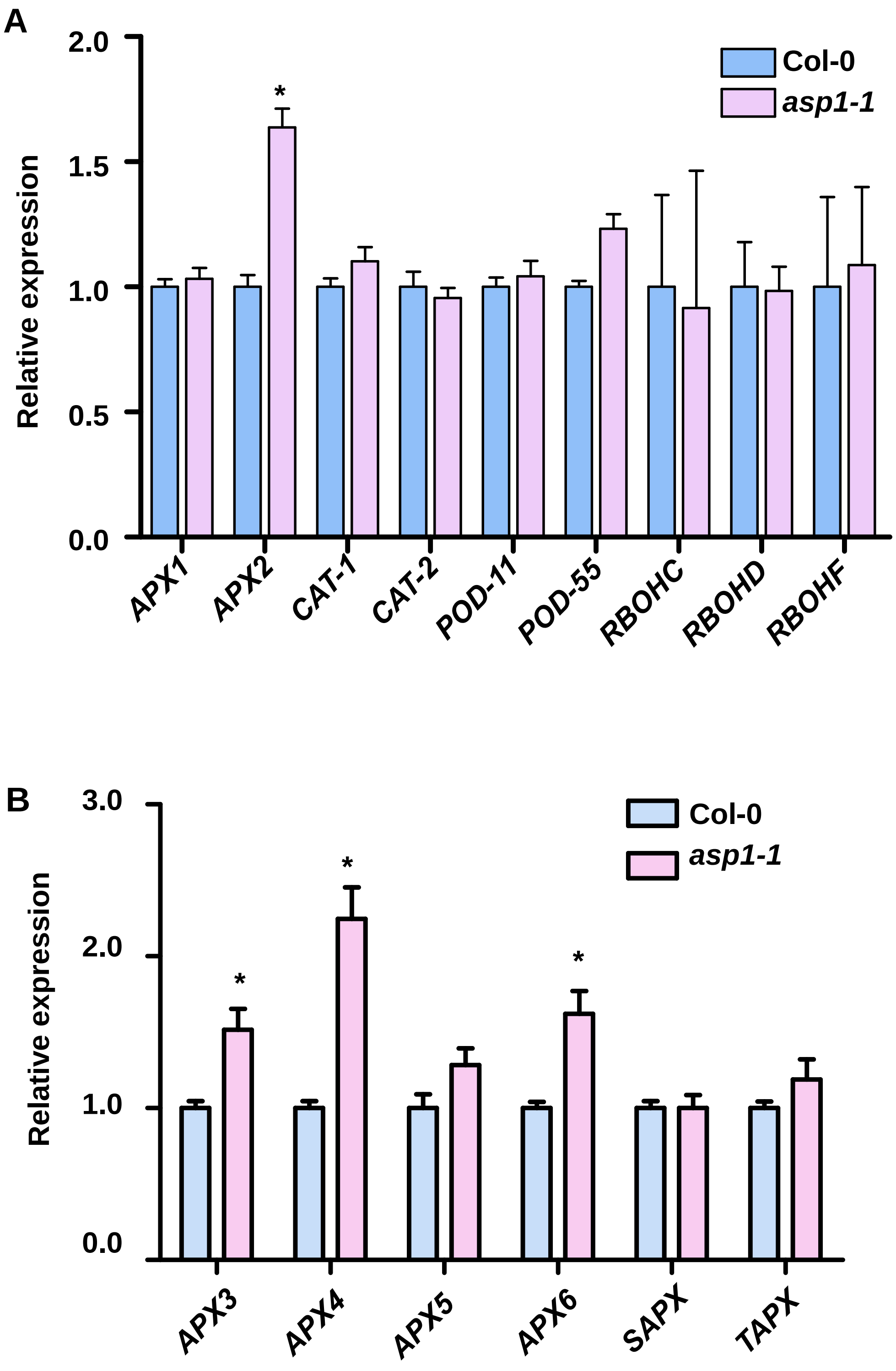

Figure S2. Quantitative real-time PCR was utilized to assess the comparative transcript levels of ROS generation and scavenging genes. The transcript levels of *APX2*, *APX3*. *APX5 and APX6* were enhanced in the *asp1* mutant. The results are presented as means ± standard error (n = 3), with statistical significance denoted by *$p < 0.05$.

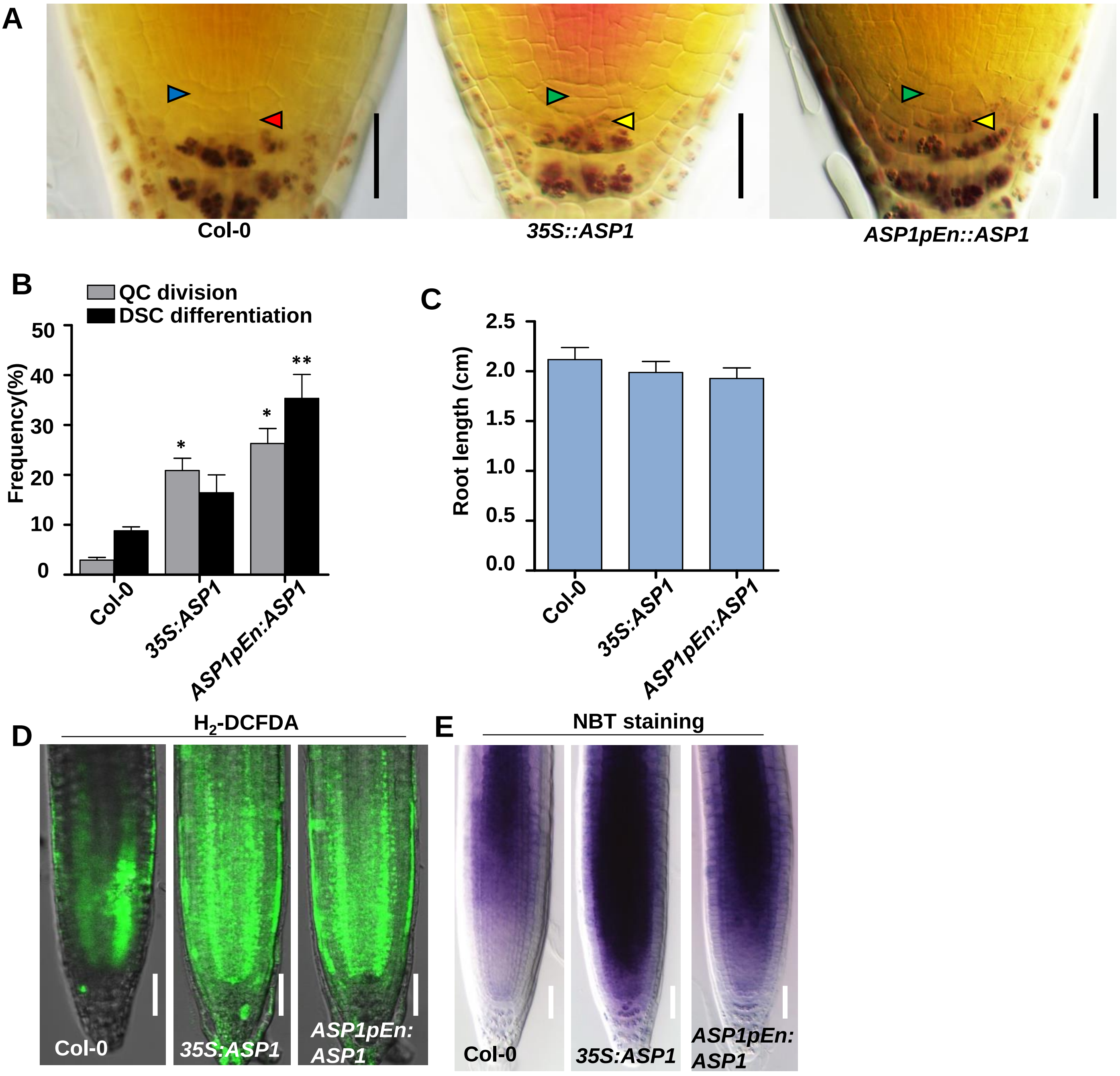

A
Col-0
35S::ASP1
ASP1pEn::ASP1
B
QC division
DSC differentiation
Frequency(%)
50
40
30
20
10
0
*
*
**
Col-0
35S:ASP1
ASP1pEn:ASP1
C
Root length (cm)
2.5
2.0
1.5
1.0
0.5
0.0
Col-0
35S:ASP1
ASP1pEn:ASP1
D
H2-DCFDA
Col-0
35S:ASP1
ASP1pEn:
ASP1
E
NBT staining
Col-0
35S:ASP1
ASP1pEn:
ASP1

Figure S3. The increased ROS concentration observed in the *ASP1*-OE line stimulates the division of QC and differentiation of DSC. (A) Lugol staining of 5-d-old Col, *35S:ASP1* and *ASP1pEn:ASP1* roots. The regular QC cells were represented by blue arrow , the undifferentiated distal stem cells were represented by red arrow, the divided QC cells were represented by green arrows, and the differentiated distal stem cells were represented by yellow arrows. The scale bars measure 50 micrometers. (B) Quantification of divided QC cells and differentiated distal stem cells. The results are presented as mean values with standard error (n = 100), where statistical significance is denoted as * for $p < 0.05$, ** for $p < 0.01$. (C) The length of primary root of five-day-old Col, *35S:ASP1* and *ASP1pEn:ASP1* . The results are presented as mean values with standard error (n = 30). (D) Fluorescence examination was conducted on H_2-DCFDA in root tips of Col, *35S::ASP1* and *ASP1pEn::ASP1*. (E) NBT staining was performed on seedlings aged 5 days, with scale bars indicating a length of 50 micrometers.

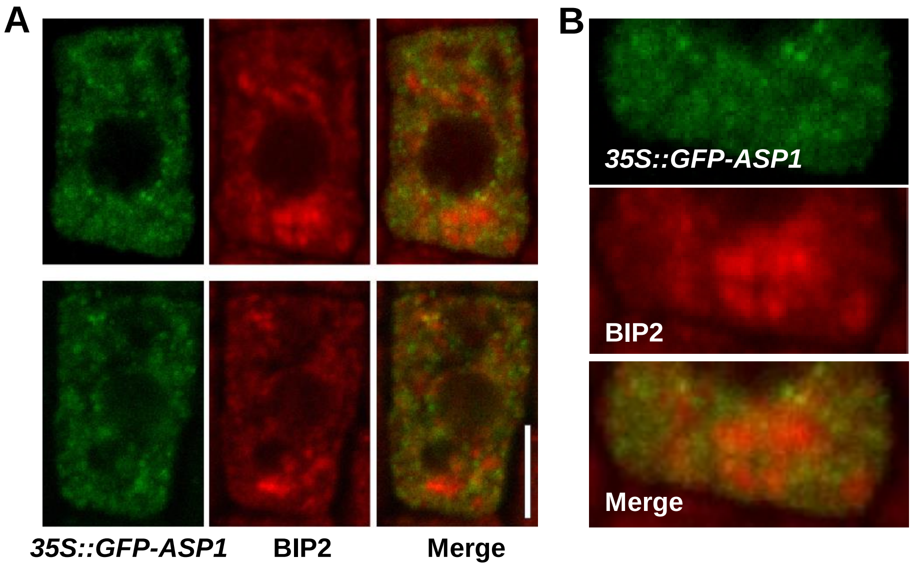

Figure S4. The subcellular distribution of ASP1 was examined, revealing minimal overlap between the signal of *GFP-ASP1* and the endoplasmic reticulum (ER) marker BIP2. The scale bar in the image represents a length of 5 μm.

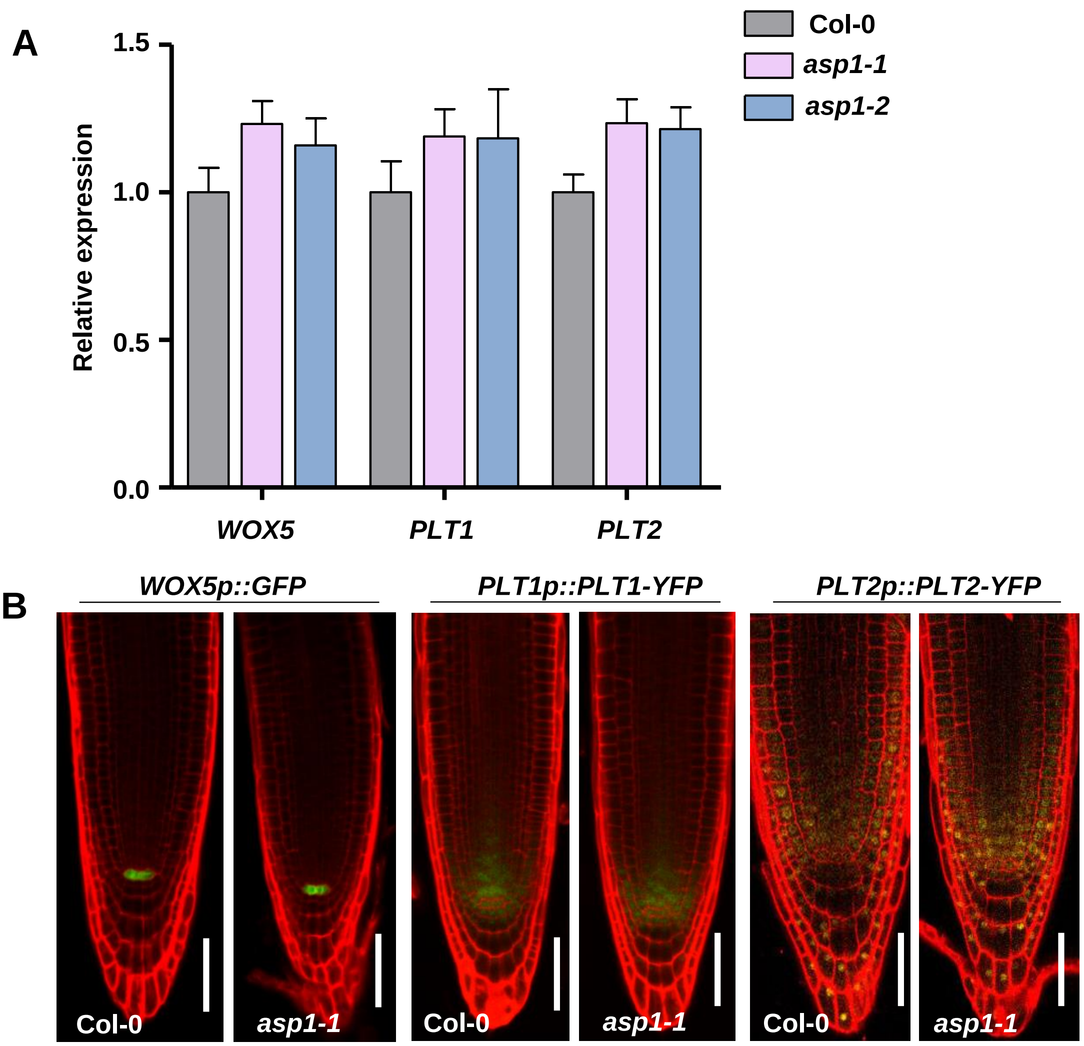

Figure S5. Expression of *WOX5*, *PLT1*, *PLT2* in *asp1* mutants did not show a statistically significant variance compared to those observed in the Col. (A) Quantitative real-time PCR was utilized to assess the comparative transcript levels of *WOX5*, *PLT1* and *PLT2* in Col and *asp1*. The results are presented as means ± standard error (*n*=3). (B) Fluorescence examination was conducted on *WOX5p::GFP*, *PLT1p::PLT1-YFP*, and *PLT2p::PLT2-YFP* in root tips of Col and *asp1*. The scale bars utilized in the analysis were set at 50 μm.
